# Contribution of p53 in sensitivity to EGFR tyrosine kinase inhibitors in non-small cell lung cancer

**DOI:** 10.1038/s41598-021-99267-z

**Published:** 2021-10-04

**Authors:** Sangyong Jung, Dong Ha Kim, Yun Jung Choi, Seon Ye Kim, Hyojeong Park, Hyeonjeong Lee, Chang-Min Choi, Young Hoon Sung, Jae Cheol Lee, Jin Kyung Rho

**Affiliations:** 1 Department of Biomedical Sciences, Asan Medical Center, AMIST, University of Ulsan College of Medicine, Seoul, 05505 Republic of Korea; 2 Asan Institute for Life Sciences, Asan Medical Center, University of Ulsan of Medicine, Seoul, 05505 Republic of Korea; 3 Department of Pulmonology and Critical Care Medicine, Asan Medical Center, University of Ulsan College of Medicine, Seoul, 05505 Republic of Korea; 4 Department of Convergence Medicine, Asan Medical Center, University of Ulsan College of Medicine, 88, Olympic-ro 43-gil, Songpa-gu, Seoul, 05505 Republic of Korea; 5 Department of Oncology, Asan Medical Center, University of Ulsan College of Medicine, 88, Olympic-ro 43-gil, Songpa-gu, Seoul, 05505 Republic of Korea

**Keywords:** Cell biology, Molecular biology, Cancer, Cancer therapy, Targeted therapies

## Abstract

The emergence of resistance to epidermal growth factor receptor (EGFR) tyrosine kinase inhibitors (TKIs) in non-small cell lung cancer (NSCLC) with activating EGFR mutations is a major hindrance to treatment. We investigated the effects of p53 in primary sensitivity and acquired resistance to EGFR-TKIs in NSCLC cells. Changes in sensitivity to EGFR-TKIs were determined using p53 overexpression or knockdown in cells with activating EGFR mutations. We investigated EMT-related molecules, morphologic changes, and AXL induction to elucidate mechanisms of acquired resistance to EGFR-TKIs according to p53 status. Changes in p53 status affected primary sensitivity as well as acquired resistance to EGFR-TKIs according to cell type. Firstly, p53 silencing did not affect primary and acquired resistance to EGFR-TKIs in PC-9 cells, but it led to primary resistance to EGFR-TKIs through AXL induction in HCC827 cells. Secondly, p53 silencing in H1975 cells enhanced the sensitivity to osimertinib through the emergence of mesenchymal-to-epithelial transition, and the emergence of acquired resistance to osimertinib in p53 knockout cells was much slower than in H1975 cells. Furthermore, two cell lines (H1975 and H1975/p53^KO^) demonstrated the different mechanisms of acquired resistance to osimertinib. Lastly, the introduction of mutant p53-R273H induced the epithelial-to-mesenchymal transition and exerted resistance to EGFR-TKIs in cells with activating EGFR mutations. These findings indicate that p53 mutations can be associated with primary or acquired resistance to EGFR-TKIs. Thus, the status or mutations of p53 may be considered as routes to improving the therapeutic effects of EGFR-TKIs in NSCLC.

## Introduction

Epidermal growth factor receptor-tyrosine kinase inhibitors (EGFR-TKIs) are widely used as molecularly targeted drugs for non-small cell lung cancer (NSCLC) harboring EGFR-activating mutations^1–3^. Although the emergence of acquired resistance to drugs is a growing problem, a variety of mechanisms of acquired resistance to EGFR-TKIs have been well defined. To date, several major mechanisms of acquired resistance have been reported, including secondary mutation of the *EGFR* gene^4,5^, amplification of the *MET* gene^6^, AXL activation^7^, and epithelial-to-mesenchymal transition (EMT)^6,8^, and effective pharmaceutical agents circumventing these mechanisms are being developed.

The p53 protein is one of the tumor suppressor genes that regulate the expression of various target genes involved in apoptosis, cell-cycle arrest, DNA repair, senescence, angiogenesis, and metastasis^9^. However, the p53 pathway is often mutated in cancer^10^, and mutations or deletions of the *p53* gene are present in approximately 50% of all human cancers^11^. The status of p53 is an important factor influencing anti-cancer drug responses. Although responses differ depending on whether mutations cause a loss of wild-type p53 or a gain or loss of mutant p53, p53 mutations or deletions have been linked to drug resistance in acute lymphoblastic leukemia^12^, melanoma^13^, osteosarcoma^14^, and breast cancer^15^, as well as ovarian and testicular cancers^16,17^.

Mutations of p53 occur in about 30–40% of NSCLC patients and in patients with smoking-associated NSCLC^18^. NSCLC patients with mutant p53 generally have more aggressive disease, increased rates of resistance to chemotherapy, and shorter survival^19,20^. The association between p53 mutations and deletions with responsiveness to EGFR-TKIs has been confirmed by many studies^21–26^. There have been reports of decreased sensitivity to EGFR-TKIs among patients with concomitant EGFR and p53 mutations^22,23,26^. Our previous study showed that wild-type p53 is needed for gefitinib-induced apoptosis and leads to increased sensitivity to EGFR-TKIs through the enhancement of Fas/FasL-mediated signaling^25^. Moreover, Canale et al. reported that p53 mutations, especially exon-8 mutations, reduced responsiveness to EGFR-TKIs and worsen prognosis in EGFR-mutated NSCLC patients, mainly those harboring exon 19 deletions^27^. Although many studies have shown that p53 mutions are associated with resistance to EGFR-TKIs, the mechanisms underlying p53 mutation-mediated resistance to EGFR-TKIs are unclear. Additionally, it is unknown whether p53 mutations affect acquired resistance to EGFR-TKIs.

We investigated the role of p53 in NSCLC cells harboring mutant EGFR using p53 overexpression or knockout to determine sensitivity to EGFR-TKIs. We analyzed whether the loss of p53 or mutant p53 affects the sensitivity or acquired resistance to EGFR-TKIs.

## Results

### The effects of p53 on sensitivity to EGFR-TKIs in PC-9 cells

**Figure 1:**
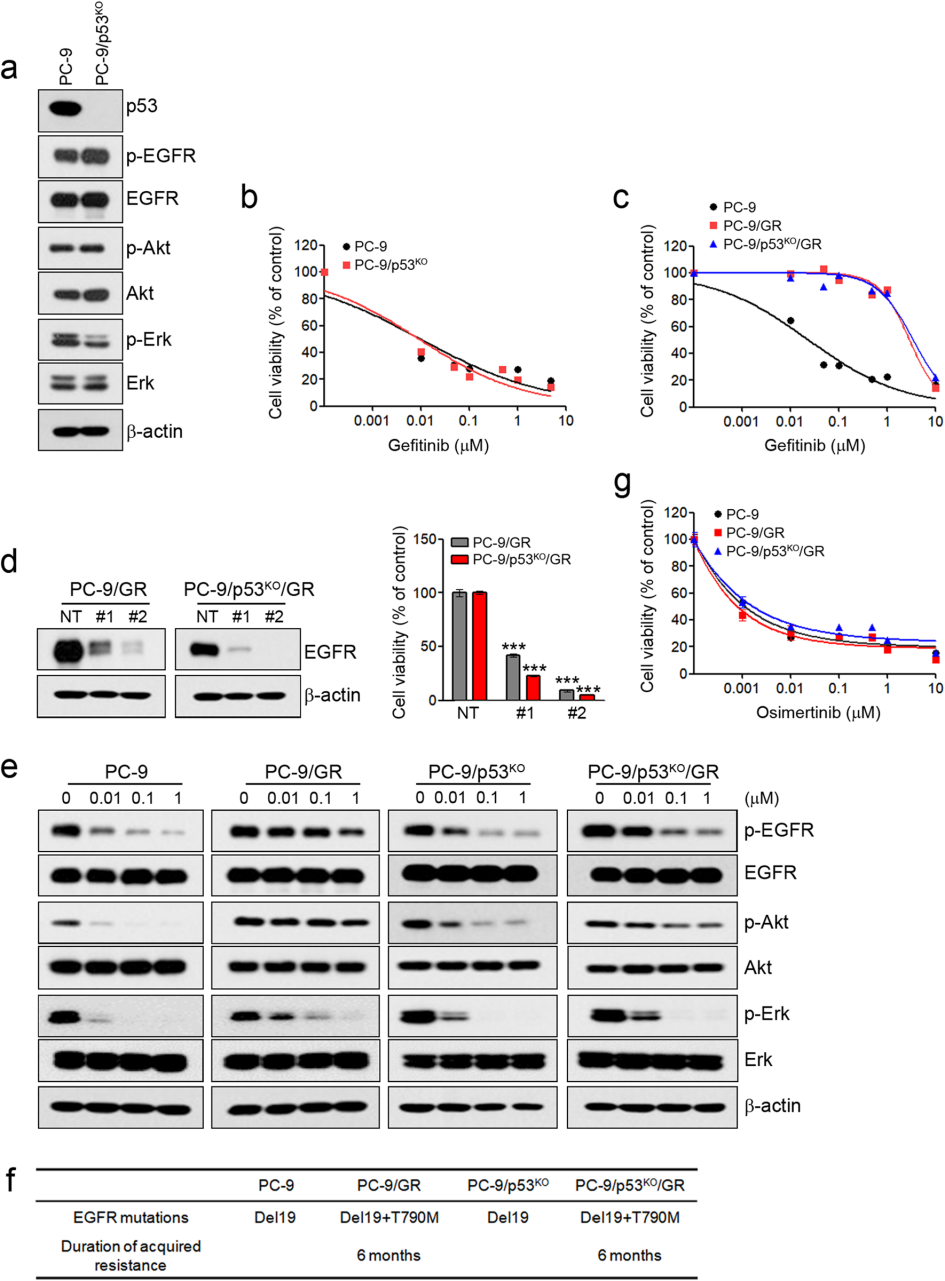
The effects of p53 on sensitivity to EGFR-TKIs in PC-9 cells. Endogenous p53 was silenced using a Crisper/Cas9 knockout system in PC-9 cells. (**a**) The indicated protein levels were analyzed by immunoblotting. (**b**,**c**) Two gefitinib-resistant cells (PC-9/GR and PC-9/p53^KO^/GR) were established as described in the Materials and Methods section. Cells were treated with the indicated doses of gefitinib for 72 h, and cell viability was determined using MTT assays. (**d**) Lentiviral constructs containing the negative control (NT) and EGFR shRNAs were introduced into the indicated cells, and EGFR suppression was confirmed by immunoblotting. Cell viability was measured by cell counting. (**e**) Cells were treated with the indicated doses of gefitinib for 6 h, and EGFR-related signaling proteins were analyzed by immunoblotting. (**f**) Cells were treated with the indicated doses of osimertinib for 72 h, and cell viability was determined using MTT assays. (**g**) EGFR mutations were measured by the PNAClamp™. ***p < 0.0005 compared with negative control shRNAs.

To evaluate the effects of p53 in primary sensitivity and acquired resistance to EGFR-TKIs in NSCLC cells with activating EGFR mutations, we generated p53 knockout cells, including PC-9 (EGFR-Del19, p53-R248Q), HCC827 (EGFR-Del19, p53-v218del), and H1975 (EGFR-L858R + T790M, p53-R273H), using CRISPR-Cas9 KO plasmids targeting the *p53* gene. Firstly, p53 knockout in PC-9 cells did not lead to significant changes in the activity of EGFR-related signaling pathways, and it did not affect the sensitivity to gefitinib (Fig. 1a,b). Next, we established cells with acquired resistance to gefitinib using PC-9 and PC-9/p53^KO^ cells (Fig. 1c). Two resistant cells (PC-9/GR and PC-9/p53^KO^/GR) exhibited acquired resistance to gefitinib during the same period (data not shown). To elucidate the mechanisms of acquired resistance to gefitinib, we first confirmed EGFR dependency. The treatment of EGFR shRNAs led to a reduction of EGFR expression and the inhibition of cell growth (Fig. 1d). However, we found that the activities of EGFR-related signaling pathways were maintained in the presence of gefitinib, unlike that observed in parental cells (Fig. 1e). We previously demonstrated that the emergence of T790M mutations was associated with acquired resistance to gefitinib or erlotinib in PC-9 cells^28^. Consistent with previous studies, T790M mutations were found in two resistant cells (Fig. 1f). Furthermore, these resistant cells were sensitive to osimertinib (Fig. 1g). These results demonstrate that the p53 status in PC-9 cells does not affect primary or acquired resistance to EGFR-TKIs.

### Silencing of p53 leads to primary resistance to EGFR-TKIs in HCC827 cells

**Figure 2:**
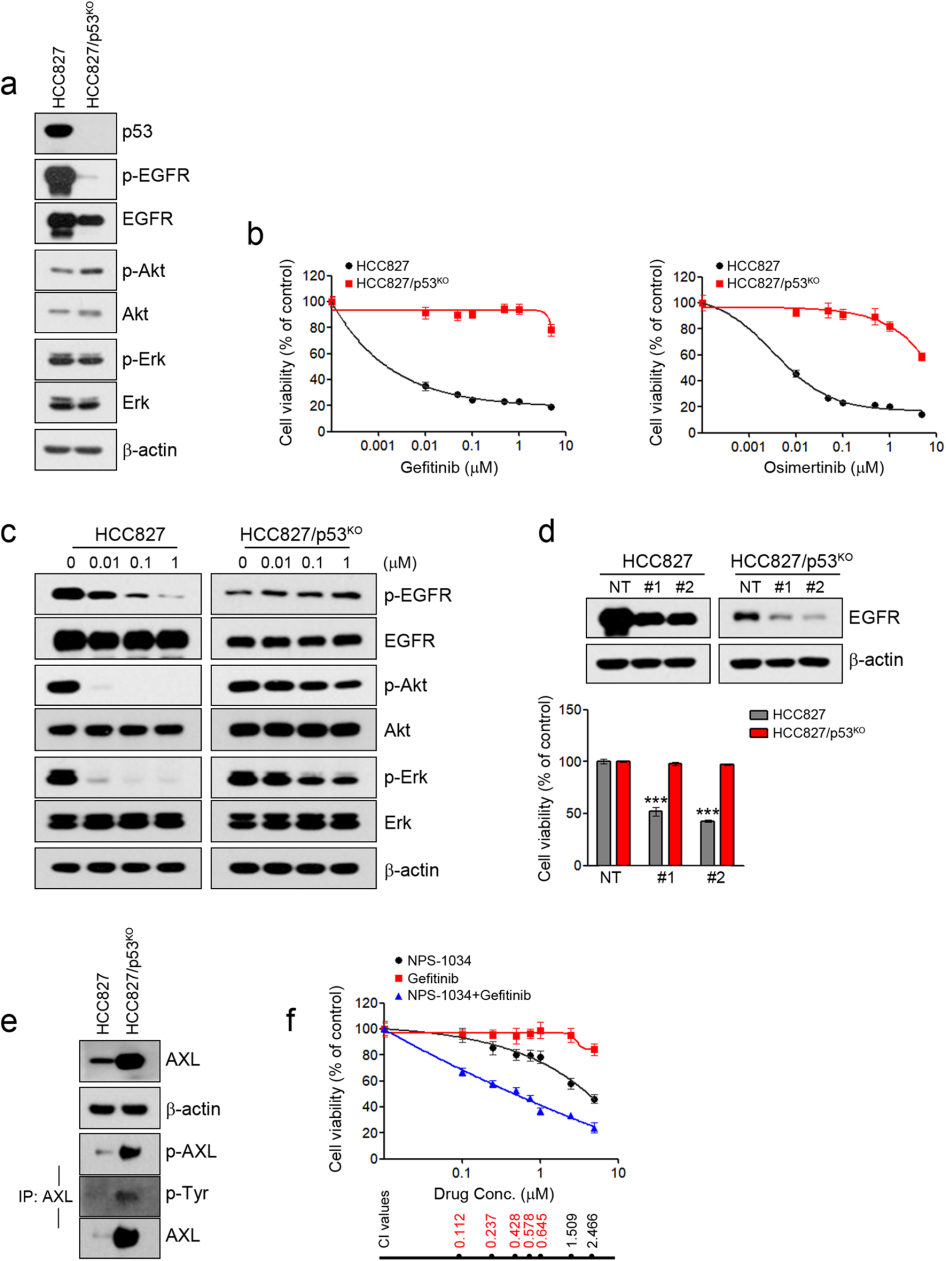
The effects of p53 on sensitivity to EGFR-TKIs in HCC827 cells. Endogenous p53 was silenced using a Crisper/Cas9 knockout system in HCC827 cells. (**a**) The indicated protein levels were analyzed by immunoblotting. (**b**) Cells were treated with the indicated doses of gefitinib or osimertinib for 72 h, and cell viability was determined using MTT assays. (**c**) Cells were treated with the indicated doses of gefitinib for 6 h, and EGFR-related signaling proteins were analyzed by immunoblotting. (**d**) Lentiviral constructs containing the negative control (NT) and EGFR shRNAs were introduced into the indicated cells, and EGFR suppression was confirmed by immunoblotting. Cell viability was measured by cell counting. (**e**) Lysates were immunoprecipitated with an anti-AXL antibody and immunoblotted with the indicated antibodies. (**f**) HCC827/p53KO cells were treated with gefitinib, NPS-1034 or a combination of the two drugs for 72 h. The combined effects were measured using the MTT assay. ***p < 0.0005 compared with negative control shRNAs.

Although p53 did not affect the sensitivity to EGFR-TKIs in PC-9 cells, p53 silencing in HCC827 cells led to a reduction of activity and expression of EGFR as well as resistance to EGFR-TKIs (Fig. 2a,b). Gefitinib treatment could effectively inhibit EGFR activation in HCC827 cells, but levels of phosphorylated EGFR and Akt were not decreased in HCC827/p53^KO^ cells (Fig. 2c). To determine whether these resistant cells depend on EGFR signaling for growth, we suppressed the *EGFR* gene using two different EGFR-specific shRNAs, and suppression of the EGFR protein was confirmed by immunoblotting. The growth of the resistant cells (HCC827/p53^KO^) was independent of EGFR signaling (Fig. 2d). Interestingly, RNA-seq analysis revealed the induction of AXL and GAS6 in HCC827/p53^KO^ that were associated with acquired resistance to EGFR-TKIs (data not shown)^7,29^. The induction of the activity and expression of AXL was confirmed using immunoblotting (Fig. 2e). To evaluate whether the induction of AXL was associated with acquired resistance to gefitinib in HCC827/p53^KO^ cells, we used NPS-1034, an AXL inhibitor. The combination of gefitinib and NPS-1034 led to the restoration of sensitivity to gefitinib (Fig. 2f). These results demonstrate that the silencing of p53 could induce the primary resistance to EGFR-TKIs through the enhancement of AXL.

### The p53 status affects primary or acquired resistance to osimertinib in H1975 cells

**Figure 3:**
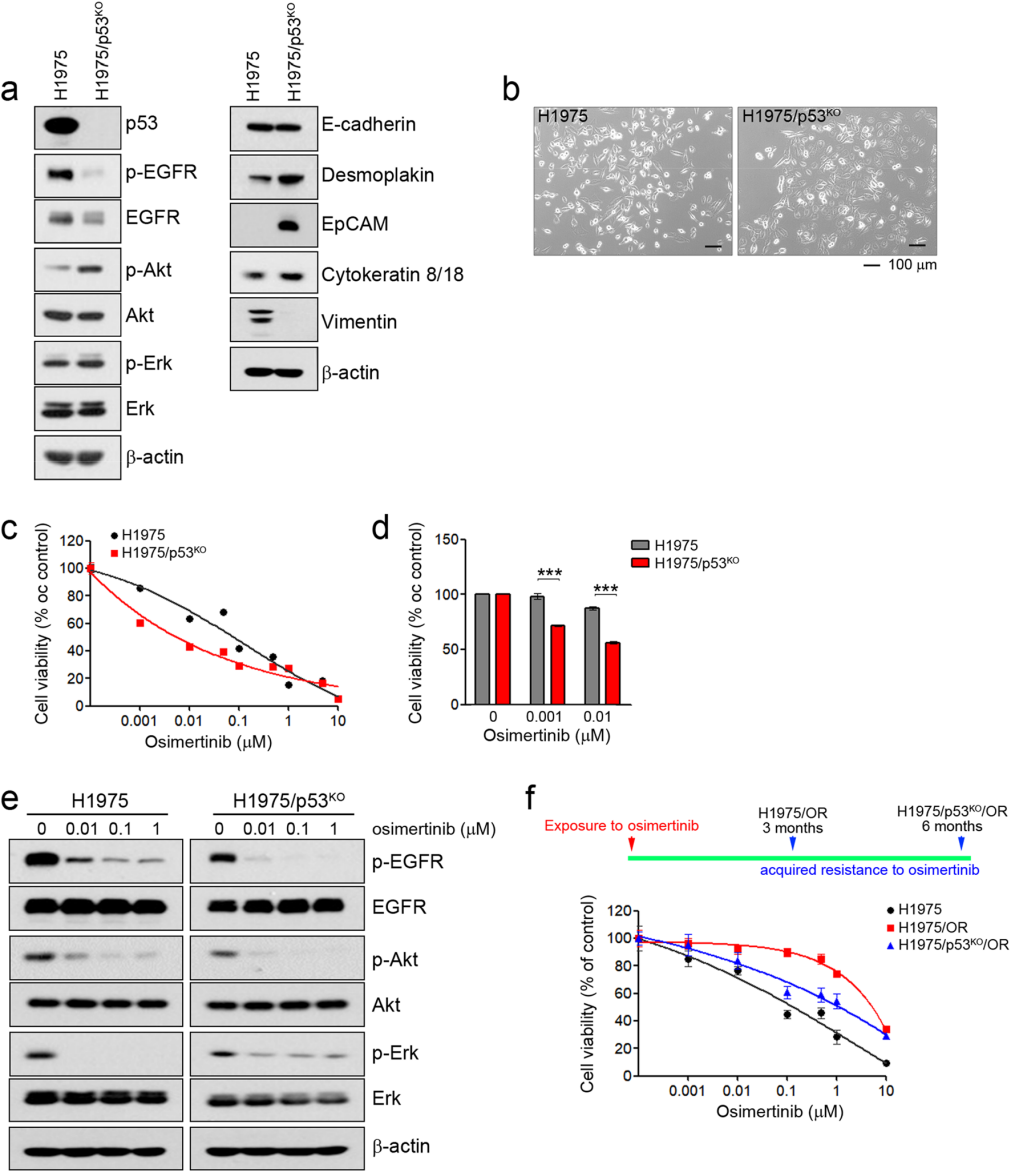
The effects of p53 on sensitivity to EGFR-TKIs in H1975 cells. Endogenous p53 was silenced using a Crisper/Cas9 knockout system in H1975 cells. (**a**) The indicated protein levels were analyzed by immunoblotting. (**b**) Cells were evaluated for morphologic changes that were consistent with MET using a light microscope. (**c**,**d**) Cells were treated with the indicated doses of osimertinib for 72 h, and cell viability was determined using MTT assays and cell counting. (**e**) Cells were treated with the indicated doses of osimetinib for 6 h, and EGFR-related signaling proteins were analyzed by immunoblotting. (**f**) Two gefitinib-resistant cells (H1975/OR and H1975/p53^KO^/OR) were established as described in the Materials and Methods section. At 3 months after drug exposure, cells were treated with the indicated doses of osimertinib for 72 h, and cell viability was determined using MTT assays. ***p < 0.0005 compared with control.

Likewise, the HCC827 results and the p53 silencing in H1975 cells led to reduced EGFR activity and expression (Fig. 3a). However, we observed morphologic differences between the H1975 and H1975/p53^KO^ cells using a light microscope. H1975 cells were round and exhibited a loss of cell–cell contacts with a mesenchymal-like phenotype, whereas H1975/p53^KO^ cells exhibited an epithelial phenotype with defined cell–cell contacts (Fig. 3b). Consistent with these morphological changes, epithelial marker proteins, including desmoplakin, EpCAM, and cytokeratin 8/18, were enhanced in H1975/p53^KO^ cells, although there was no difference in E-cadherin expression between the two cell types (Fig. 3a). Interestingly, H1975/p53^KO^ cells were more sensitive to osimertinib than H1975 cells (Fig. 3c,d). Consistent with these results, osimertinib treatment effectively inhibited EGFR-related signaling in H1975/p53^KO^ cells compared with H1975 cells (Fig. 3e).

To determine whether p53 affects acquired resistance to EGFR-TKIs, we established cells with acquired resistance to osimertinib using H1975 and H1975/p53^KO^ cells. The period time of acquisition of resistance to osimertinib showed different patterns according to p53 status. H1975/OR cells were generated 3 months following exposure to osimertinib, whereas H1975/p53^KO^/OR cells took 6 months (Fig. 3f). Additionally, the mechanisms of acquired resistance to osimertinib in H1975/OR cells were the induction of EMT in previous studies^30^. However, changes in EMT were not observed in H1975/p53^KO^/OR cells (Supplementary Fig. S1). Taken together, these results showed that p53 could affect primary sensitivity as well as the mechanisms and time of acquired resistance to EGFR-TKIs.

### Mutant p53-R273H induces EMT and leads to resistance to EGFR-TKIs

**Figure 4:**
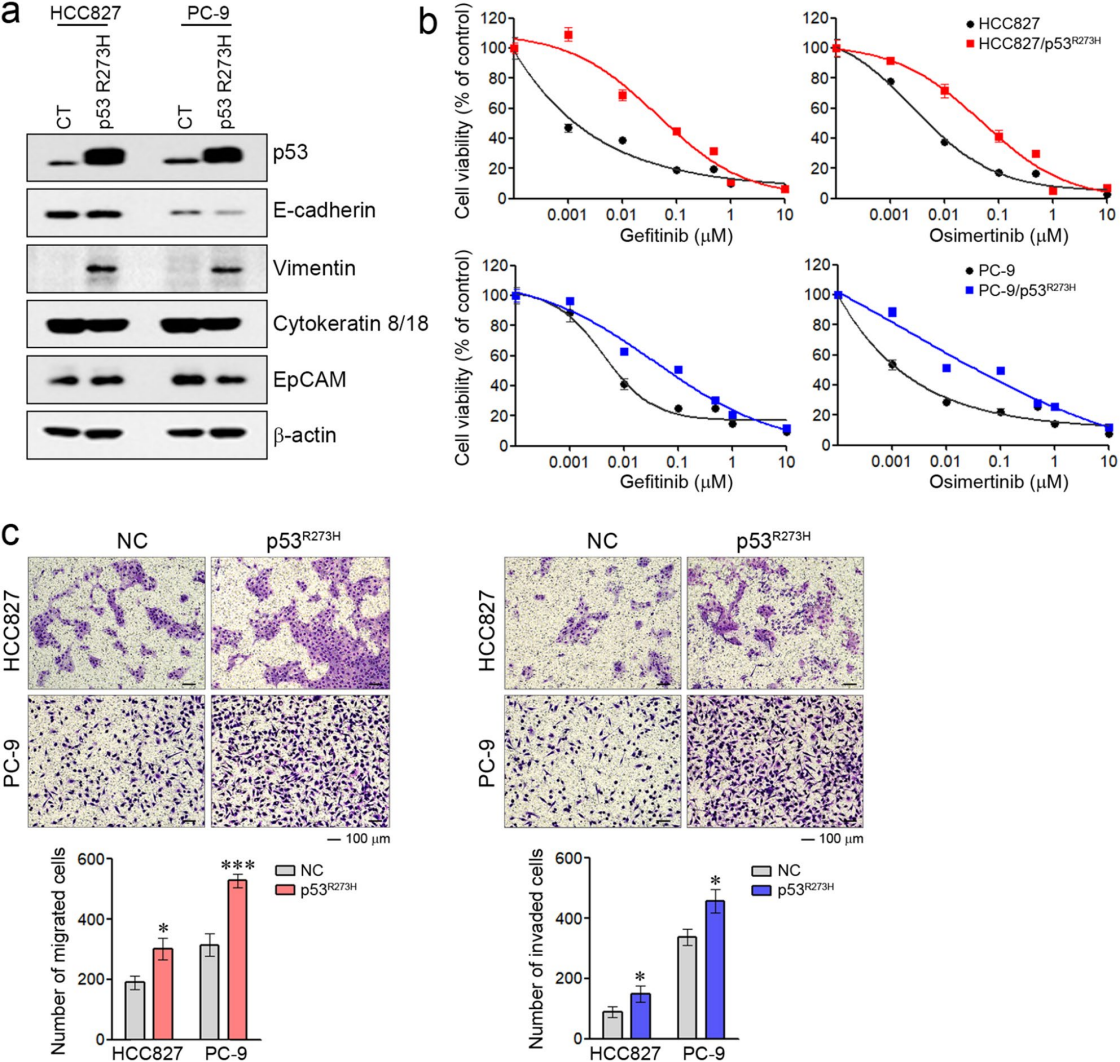
The effects of p53-R273H on sensitivity to EGFR-TKIs in NSCLC cells with activating EGFR mutations. Mutant p53-R273H was introduced into cells with activating EGFR mutations. (**a**) p53 and EMT-related protein levels were analyzed by immunoblotting. (**b**) Cells were treated with the indicated doses of gefitinib or osimertinib for 72 h, and cell viability was determined using MTT assays. (**c**) Cells were seeded onto either collagen- or Matrigel-coated polycarbonate filters to determine their migratory and invasive potentials, respectively. Cells were incubated in modified Boyden chambers for 24 h, and the cells that penetrated the filter were stained and counted using a light microscope. Experiments were conducted in triplicate. *p < 0.05; ***p < 0.0005 compared with control.

H1975 cells have mutant p53-R273H^31,32^. Some authors suggest that mutant p53-R273H gain of function induces EMT^33,34^. Thus, we investigated whether such mutant p53 affects the sensitivity to EGFR-TKIs via EMT induction in cells with activating EGFR mutations. When mutant p53-R273H was introduced into HCC827 and PC-9 cells, the induction of vimentin was observed in two cells (Fig. 4a). Although the changes of EMT-related molecules differed in the two cells, induction of the EMT-like phenotype was more evident in PC-9 cells than HCC827 via the reduction of E-cadherin, cytokeratin 8/18, EpCAM, and the induction of vimentin. EMT induction by mutant p53-R273H was also confirmed in other cells with wild-type or null p53 (Supplementary Fig. S2). Furthermore, the introduction of mutant p53-R273H led to resistance to EGFR-TKIs in two cells (Fig. 4b). Lastly, we examined migratory and invasive potential, which are considered functional hallmarks of EMT. We found that the ability to migrate and invade was significantly increased in cells that express mutant p53-R273H (Fig. 4c). Taken together, these findings suggest that the expression of mutant p53-R273H could lead to resistance to EGFR-TKIs via EMT induction.

## Discussion

Our previous study and other studies suggested that p53 is associated with primary and acquired resistance to EGFR-TKIs^21–26^. Recently, some studies have demonstrated that p53 mutations confer worse prognoses in EGFR-mutated NSCLC patients treated with EGFR-TKIs^35–37^. However, the mechanisms of primary and acquired resistance to EGFR-TKIs by mutant p53 are still unclear. To begin to address this issue, we investigated the role of mutant p53 in systems including overexpression or knockout of mutant p53. We used EGFR-mutated NSCLC cell lines, such as HCC827, PC-9, and H1975, with different types of p53 mutations. In previous studies, they were classified as PC-9 (p53-R248Q), HCC827 (p53-v218del), and H1975 (p53-R273H)^38^. Although the function of p53-v218del is unknown, p53-R248Q and p53-R273H were associated with cancer progression steps, including tumorigenesis, stemness, and metastasis, as gain-of-function mutant p53^39–41^. In our data, p53-R248Q did not affect the sensitivity and acquired resistance to EGFR-TKIs in PC-9 cells. However, the loss of p53-v218del led to primary resistance to EGFR-TKIs through AXL induction in HCC827 cells. Furthermore, p53-R273H expression directly led to EMT induction, and these phenomena were associated with primary and acquired resistance to EGFR-TKIs. Although the mechanisms of sensitivity and acquired resistance to EGFR-TKIs differed according to the type of p53 mutations, p53 mutations can affect sensitivity or acquired resistance to EGFR-TKIs in EGFR-mutated NSCLC cells.

AXL receptor tyrosine kinase is known as a promising anti-cancer target^42^. In particular, some authors suggest that AXL is associated with acquired resistance to EGFR-TKIs in EGFR-mutated NSCLC^7,29^. This resistance frequently accompanies EMT^7^. Although various studies have demonstrated the mechanisms of AXL-mediated drug resistance, the mechanisms underlying AXL upregulation are unclear. In our studies, the silencing of mutant p53-v218del led to AXL induction and subsequently resulted in resistance to EGFR-TKIs. Boysen et al. suggested that a loss of p53 function could induce AXL expression via downregulation of miR-34a in B-cell chronic lymphocytic leukemia^43^. Conversely, Vaughan et al. suggested that gain-of-function mutant p53 (R175H, R273H, and D281G) promotes AXL expression at both the RNA and protein level^44^. Thus, we are investigating the roles of p53 loss of function and gain of function to better understand AXL upregulation, although the roles can vary according to the type of mutant p53.

Accumulating evidence has revealed that EMT is correlated with a poor prognosis for NSCLC patients^45,46^ as well as acquired resistance to various chemotherapeutic agents, including EGFR-TKIs^8,30,47–49^. Some studies have demonstrated that TGF-β, IGF1R, AXL, Notch signaling, and miRNAs are involved in the EMT process^7,50–52^, although the mechanisms of EMT are complex and versatile. However, the mechanisms of EMT in acquired resistance to EGFR-TKIs are still unclear. In our study, we showed that mutant p53-R273H leads to the induction of an EMT-like phenotype. Furthermore, mutant p53-R273H played important roles in the emergence of acquired resistance to EGFR-TKIs. Cells with mutant p53-R273H appeared the induction of EMT as the mechanism of the acquired resistance to EGFR-TKIs, and the period of acquisition of resistance to EGFR-TKIs was shorter than for cells with mutant p53-R273H^KO^. Although the mechanisms of acquired resistance to EGFR-TKIs in cells with mutant p53-R273H^KO^ were not known or revealed in this study, EMT induction was not observed. Thus, mutant p53-R273H can contribute to EMT-mediated resistance and short-term of EGFR-TKIs treatment.

A few limitations of this study should be taken into consideration. Firstly, many different types of tumors show a high incidence of p53 mutation, and there are many types of p53 mutations. Although some studies have suggested that p53 mutations, especially exon-8 mutations, reduce responsiveness to EGFR-TKIs and worsen the prognosis for patients with EGFR-mutated NSCLC^27,35^, further studies are needed to determine the potentially varying effects of the different p53 mutation types. Secondly, the detailed mechanisms underlying EMT induction by mutant p53-R273H were not investigated. Some studies have suggested that the inhibition of Twist1 degradation and miR-130b-ZEB1 pathways are associated with EMT and gain-of-function p53 mutations, including p53-R273H^33,53^. Thus, further research is required to identify the specific mechanisms of action involved.

In summary, mutant p53 can affect both primary and acquired resistance to EGFR-TKIs; in particular, mutant p53-R273H is associated with resistance to EGFR-TKIs via EMT induction. Thus, the p53 status should be a consideration in efforts to improve the therapeutic efficacy of EGFR-TKIs for NSCLC.

## Methods

### Cell culture and reagents

H1975 and HCC827 cell lines were obtained from the American Type Culture Collection (Rockville, MD), and the PC-9 cell line was provided by Dr. Kazuto Nishio (National Cancer Center Hospital, Tokyo, Japan). All cells were maintained in RPMI 1640 (Invitrogen, Carlsbad, CA, USA) with 100 U/mL penicillin, 100 mg/mL streptomycin (Invitrogen), and 10% fetal bovine serum (FBS). Gefitinib, osimertinib, and NPS-1034 were purchased from Selleck Chemicals (Houston, TX, USA). We purchased 3-(4,5-dimethylthiazo-2-yl)-2,5-diphenyltetrazolium bromide (MTT) solution from Sigma (St. Louis, MO, USA).

### Establishment of the p53-knockout EGFR mutant NSCLC cell lines

Cells were transfected with CRISPR-Cas9 Knockout (KO) plasmids with target-specific guide RNA (gRNA) of the *p53* gene (LentiCRISPR v2, #52961, Addgene, Cambridge, MA, USA), which allows the insertion of the puromycin resistance gene. After 48 h, cells were selected at a concentration of 5 µg/mL puromycin, and p53 silencing was determined by immunoblotting.

### Generation of cell lines with acquired resistance to EGFR-TKIs

All resistant cells were established by chronic, repeated exposure to gefitinib or osimertinib, as reported in previous studies^28,30^. For all experiments, resistant cells were cultured in a drug-free medium for at least 1 week to eliminate gefitinib or osimertinib. Gefitinib- and osimertinib-resistant cells are referred to as PC-9/GR and H1975/OR cells, respectively.

### EGFR mutation analysis

A peptide nucleic acid (PNA)-mediated PCR clamping assay (PNACLamp EGFR Mutation Detection kit, PANAGENE Inc., Daejeon, Korea) was used to detect T790M mutations. The detection of T790M mutations was performed as previously described^54^.

### Lentivirus-mediated gene suppression or overexpression

To suppress EGFR, we used shEGFR-1 (TRCN0000195303) and shEGFR-2 (TRCN0000298822). Cells were transiently infected with shControl, shEGFR-1, or shEGFR-2 for 48 h. After infection, EGFR expression was determined by immunoblotting. Cell viability was determined by trypan blue staining using an ADAM-MC automatic cell counter (NanoEnTek, Seoul, Korea). Exogenously expressed mutant p53-R273H, pLenti6/V5-p53-R273H (plasmid #22934), was purchased from Addgene (Cambridge, MA, USA). Cells were infected with viral particles for 48 h. After infection, cells were selected using 5 μg/mL blasticidin. Mutant p53 expression was determined by immunoblotting.

### MTT assays

MTT assay was performed as previously described^55^. Briefly, cells (1 × 10^4^) were seeded in 96-well plates overnight. The indicated drugs were added in a dose dependent manner and the cells were incubated for 72 h. The combined effects of drugs were assessed by MTT assay at a 1:1 ratio of each drug. CI values were determined by using the CalcuSyn Software (Biosoft). The results represent at least three independent experiments, and the error bars signify the standard deviation from the mean.

### Invasion and migration assay

Cell migration and invasion assays were performed according to previously described method^8^. Triplicate results are expressed as mean (standard deviation).

### Immunoblotting and immunoprecipitation

Whole-cell lysates were prepared as previously described^55^. Antibodies specific for p53 (1:2000, sc-126), p-EGFR (1:1000, Tyr1173, sc-101668), EGFR (1:2000, sc-373749), p-Erk (1:1000, Thr202/Tyr204, sc-16982), Erk (1:3000, sc-94), Akt (1:3000, sc-5298), AXL (1:1000, sc-1096), E-cadherin (1:1000, sc-71008), EpCAM (1:1000, sc-71059), desmoplakin (1:1000, sc-390975), cytokeratin-8/18 (1:1000, sc-70939) were purchased from Santa Cruz Biotechnology (Santa Cruz, CA, USA); those for p-Akt (1:1000, Ser473, #4060), β-catenin (1:1000, #8480), p-AXL (1:1000, #4060), and vimentin (1:1000, #5741) were obtained from Cell Signaling Technology (Beverly, MA, USA). To assess the level of p-AXL, lysates were immunoprecipitated with an anti-AXL antibody and immunoblotted with an anti-phosphotyrosine (p-Tyr, 1:1000, sc-7020, Santa Cruz) antibody. The immunoblotting is representative of three independent experiments.

### Statistical analyses

Data are presented as the mean ± standard deviation, and *p* values were determined using unpaired *t*-tests between groups with GraphPad Prism (GraphPad Software, San Diego, CA, USA).

## Supplementary Information

Supplementary Information 1.

Supplementary Information 2.

## Data Availability

All data generated or analyzed during this study are included in this published article and the Supplementary Information.
