# Supplementary figures and images for: Contribution of p53 in sensitivity to EGFR tyrosine kinase inhibitors in non-small cell lung cancer
Source: Sci Rep. 2021 Oct 4;11:19667. doi: 10.1038/s41598-021-99267-z (PMC8490392; doi:10.1038/s41598-021-99267-z)

Fig.1a

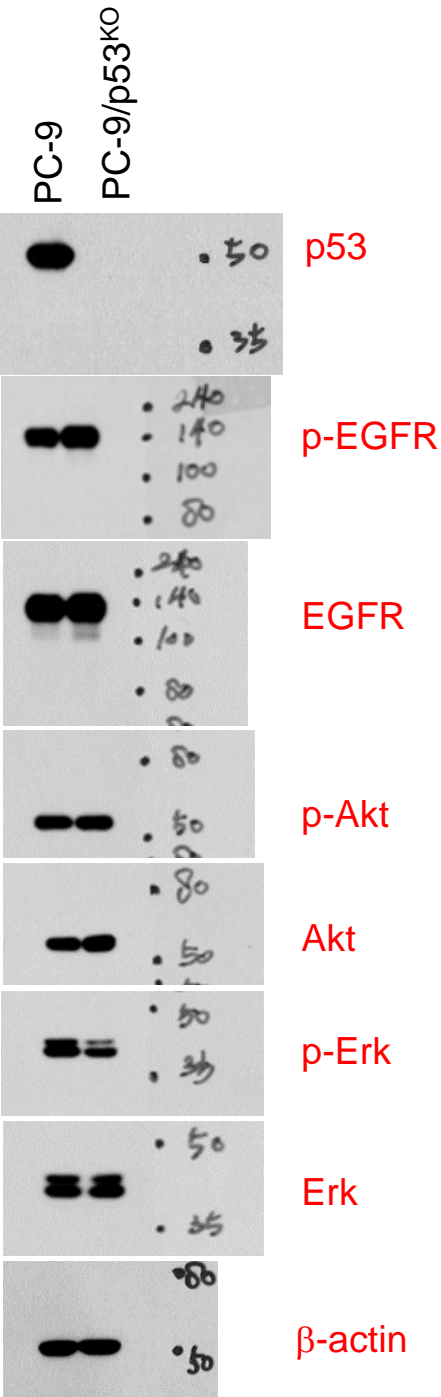

Fig. 1d

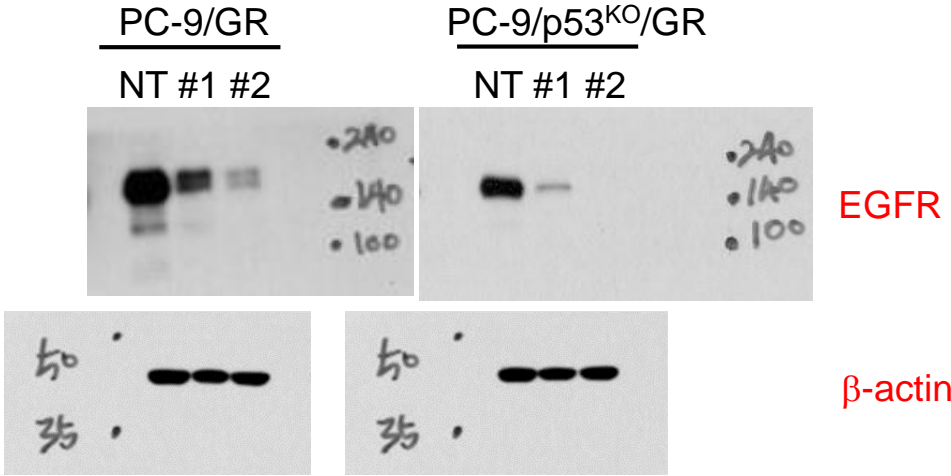

Fig.1e

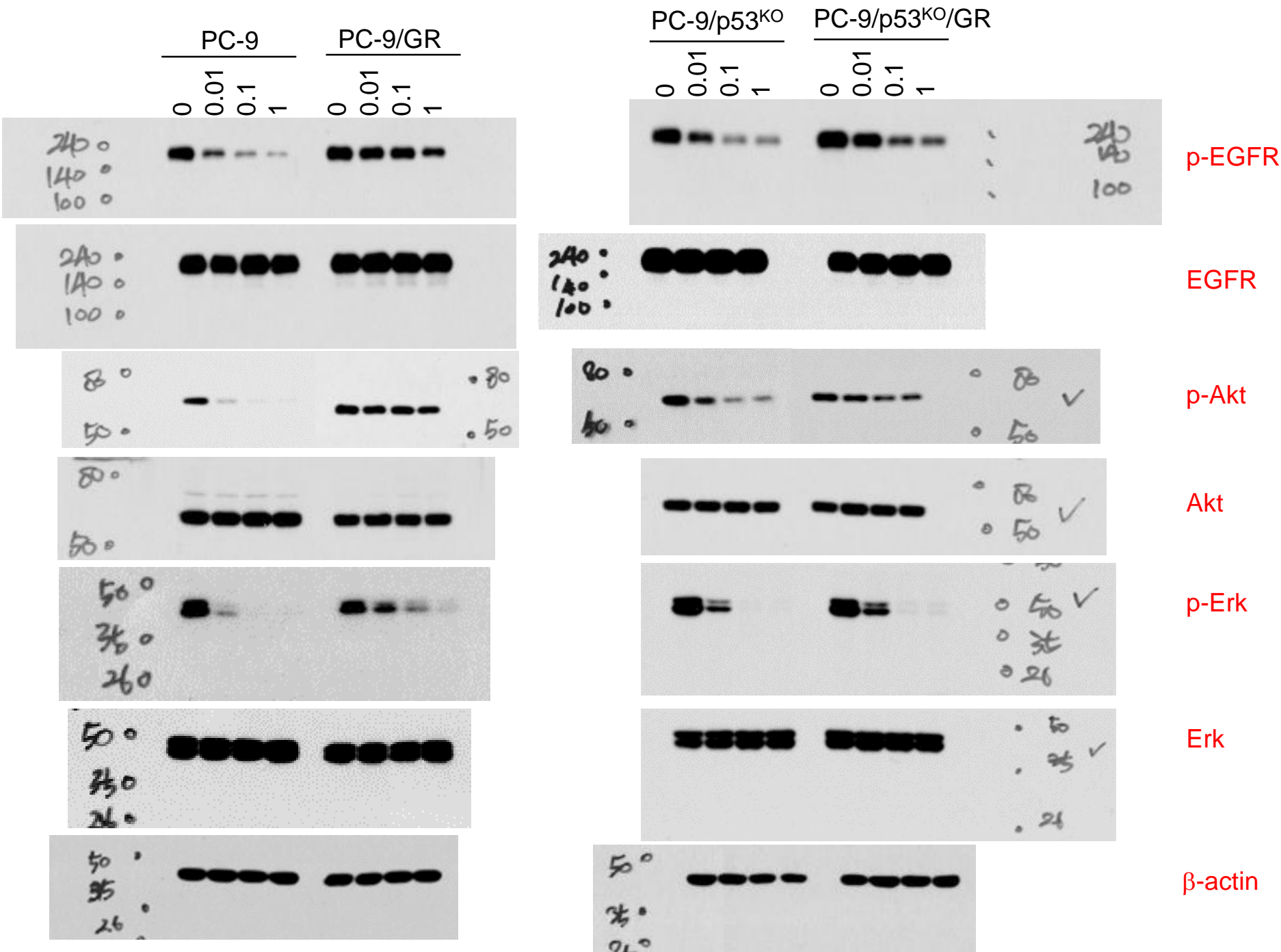

Fig. 2a

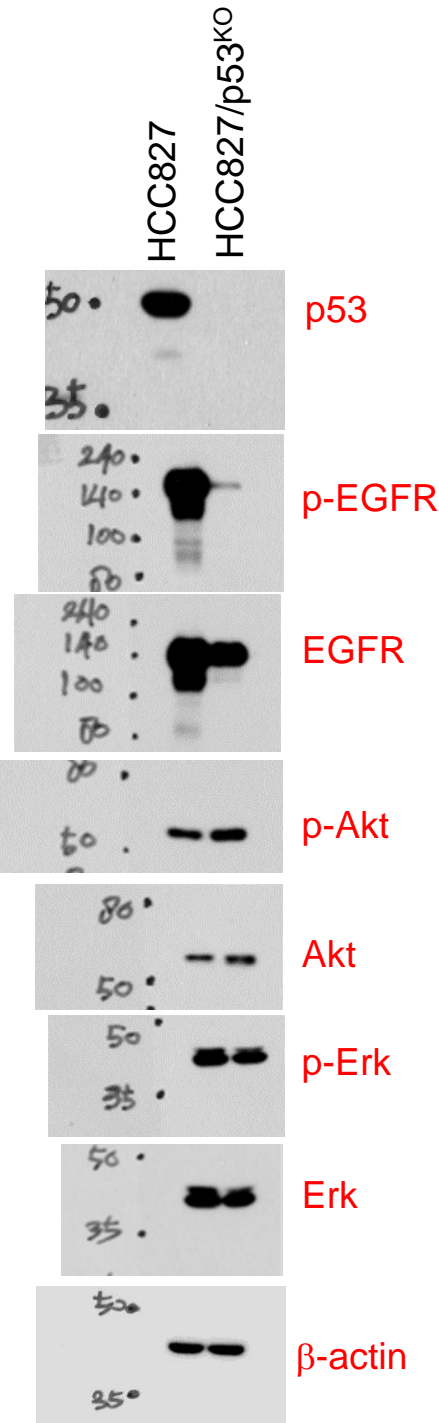

Fig. 2c

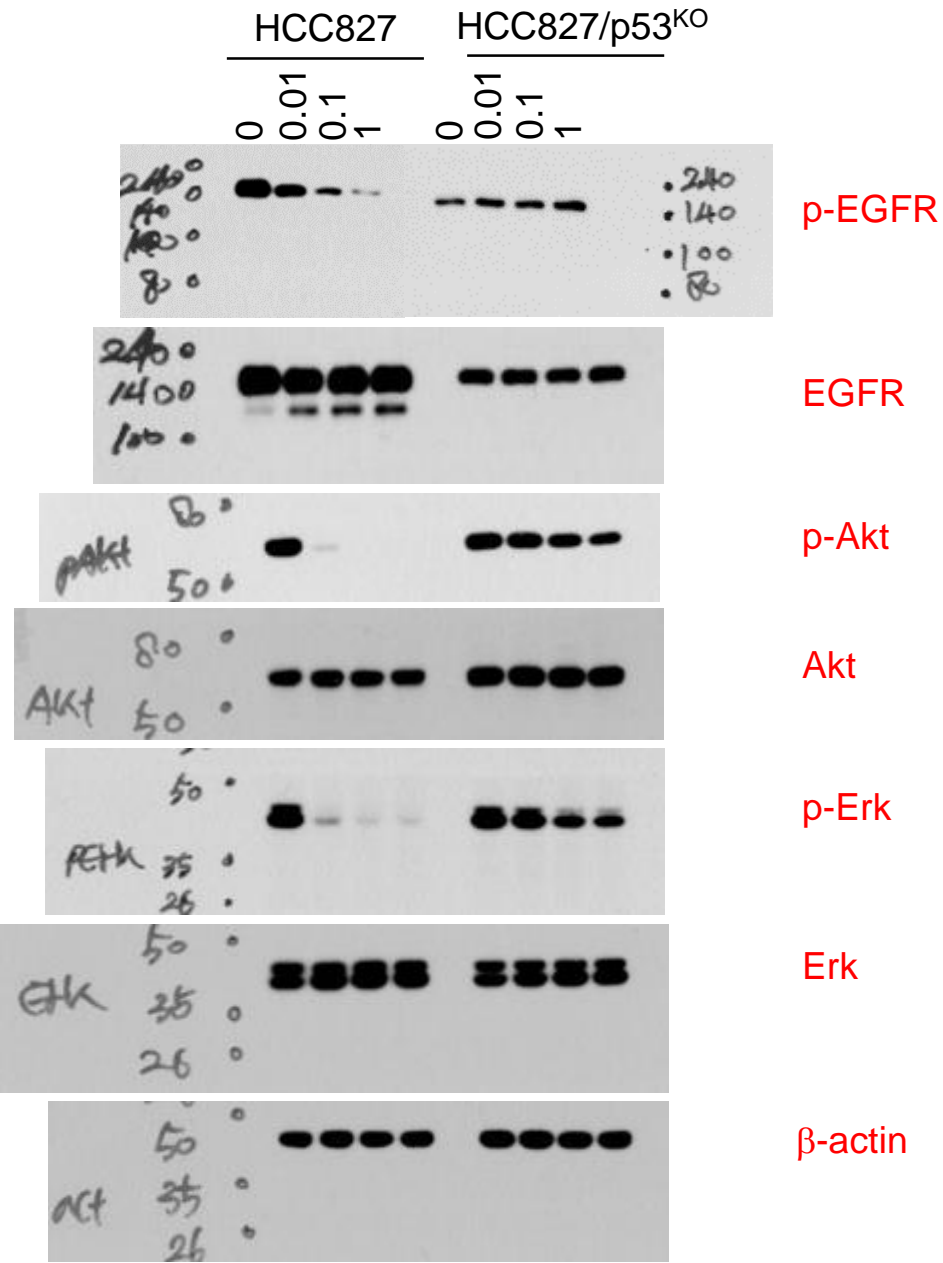

Fig. 2d

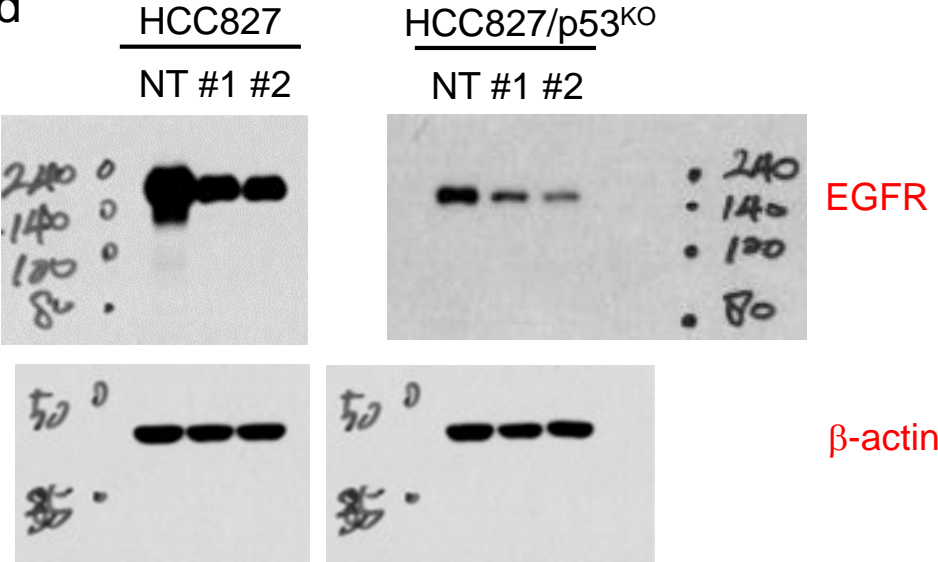

Fig. 2e

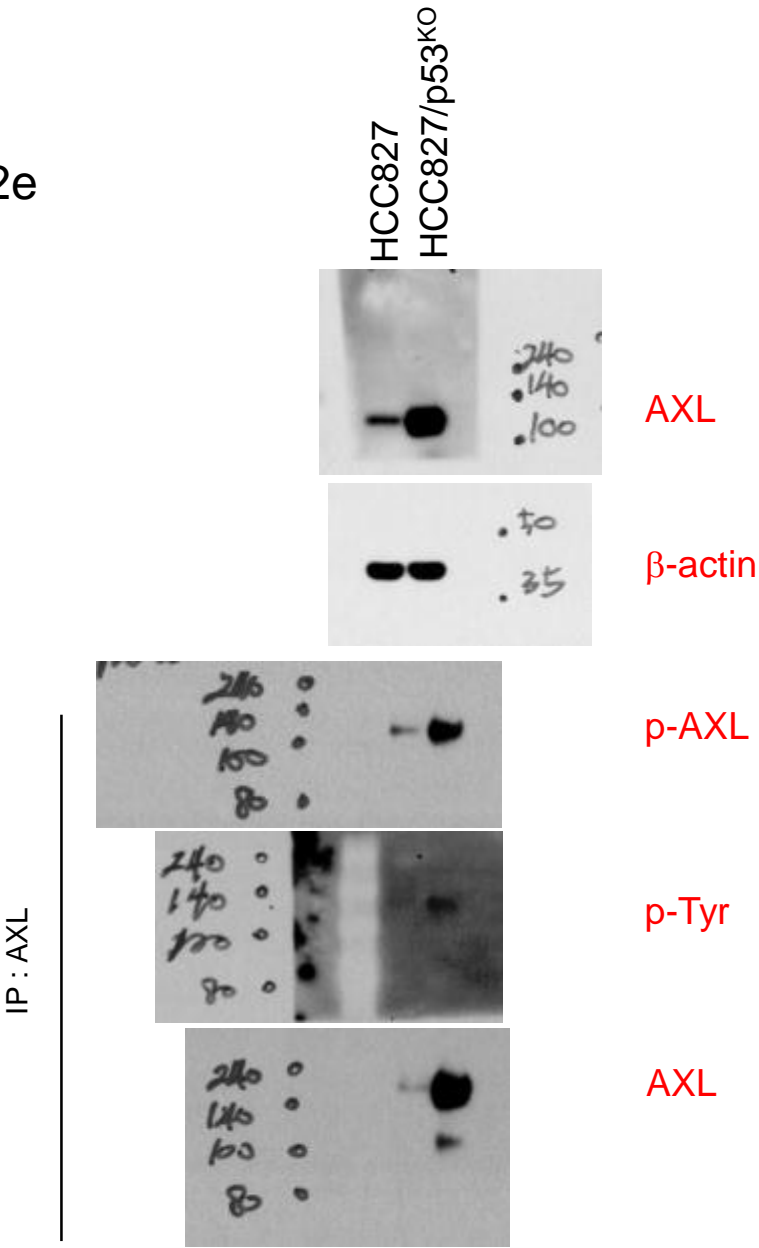

Fig. 3a

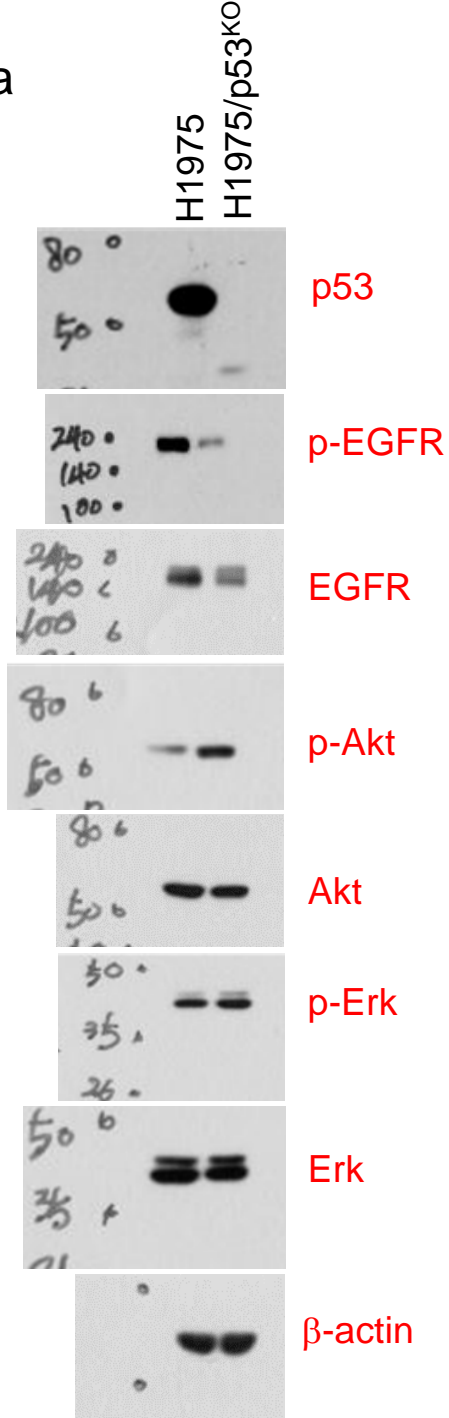

Fig. 3e

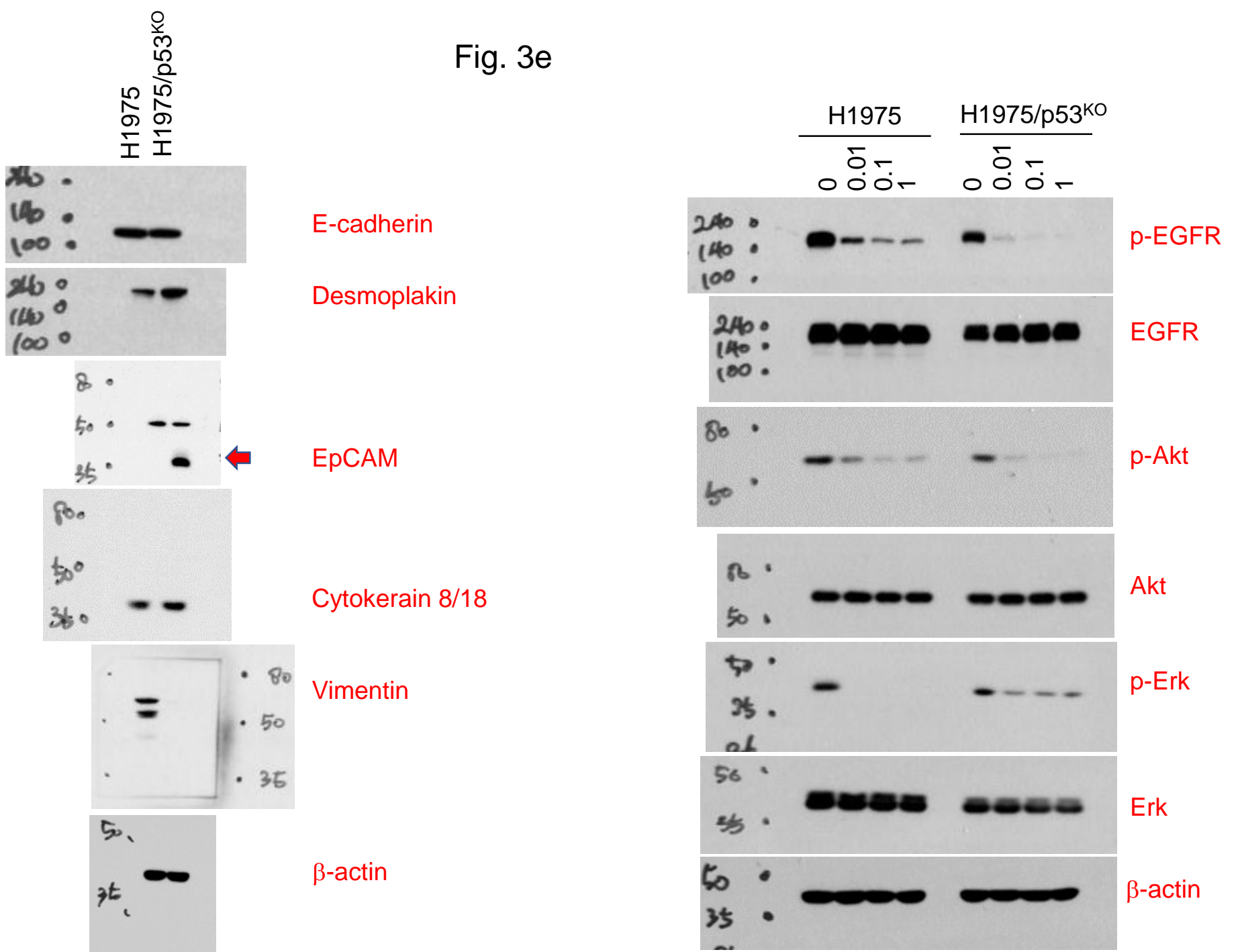

Fig. 4a

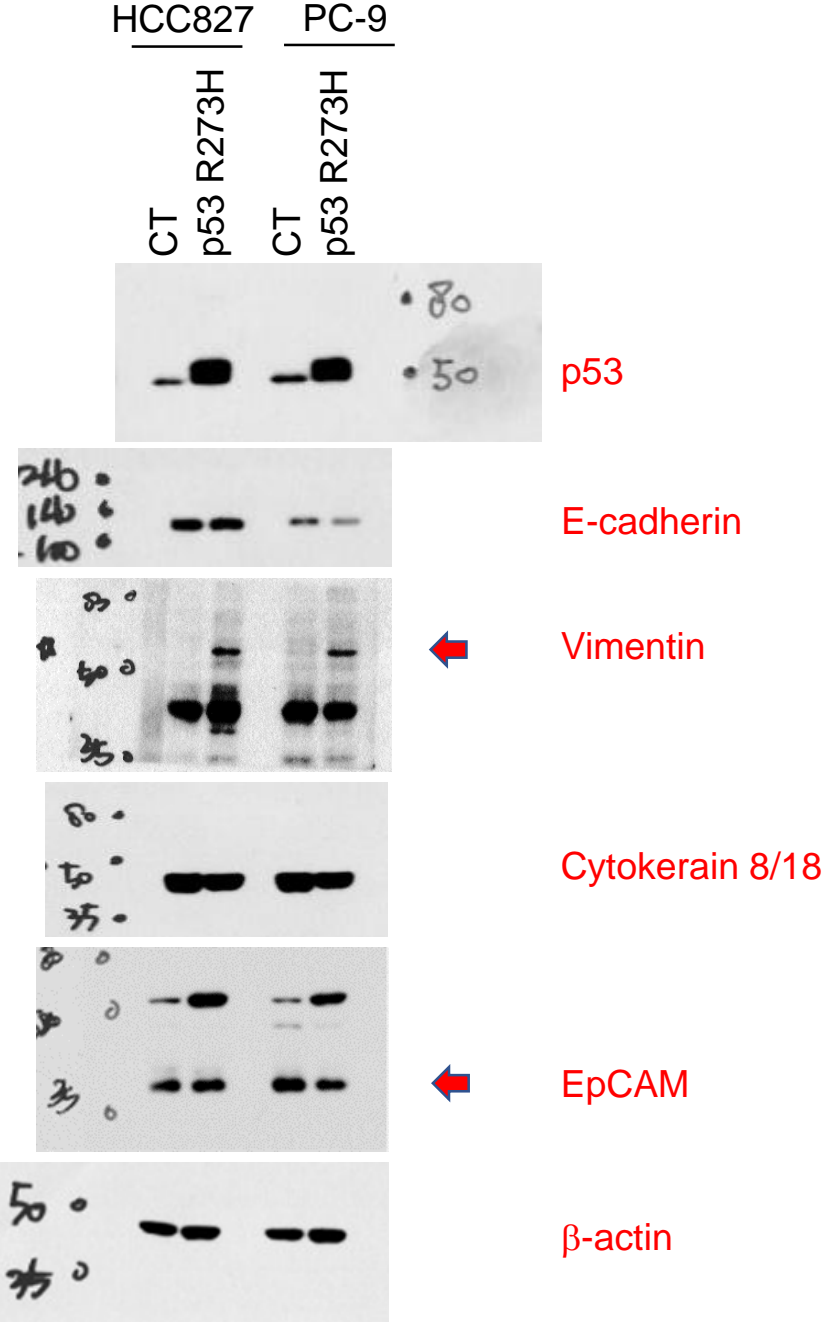

Supplemental Fig. 1c

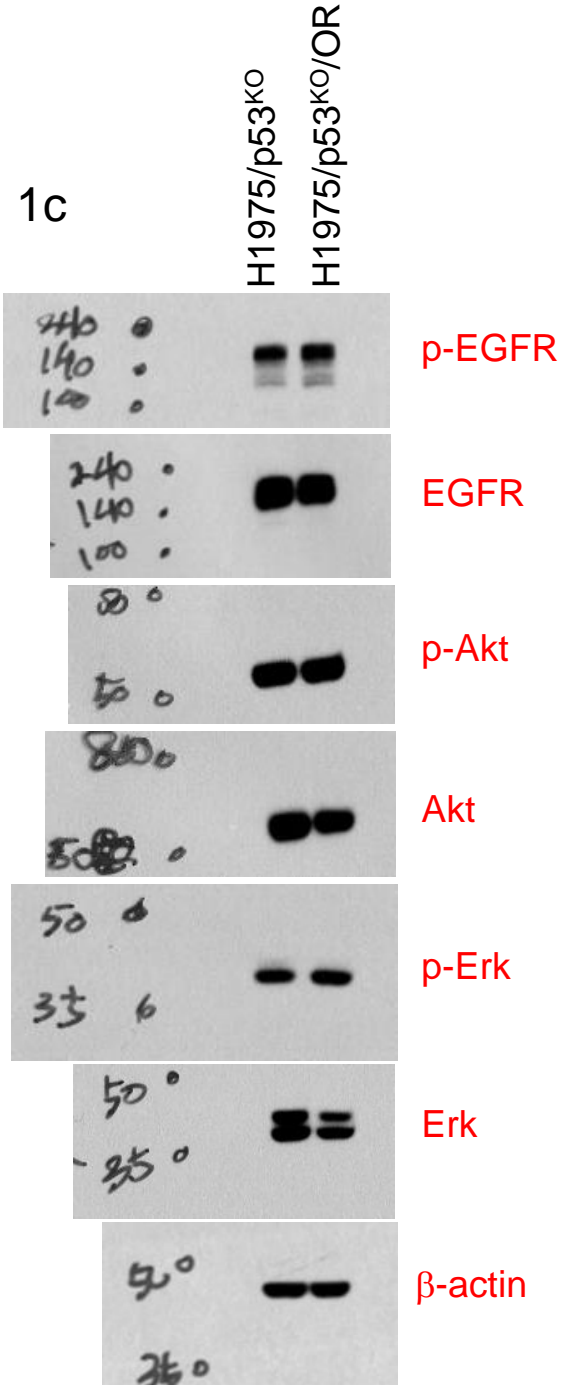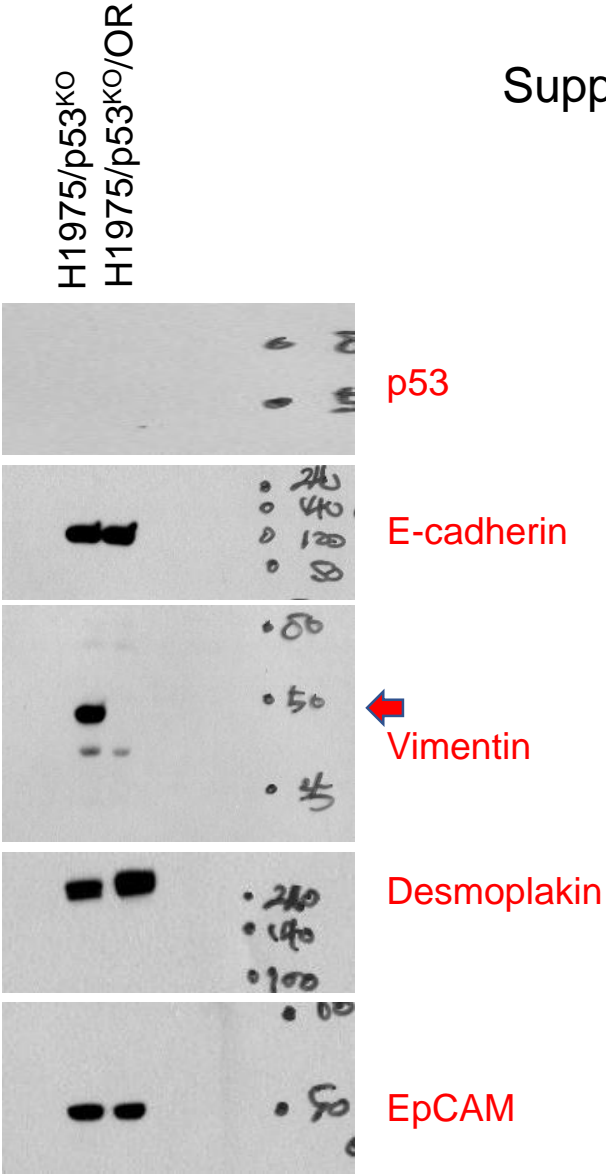

Supplemental Fig. 2

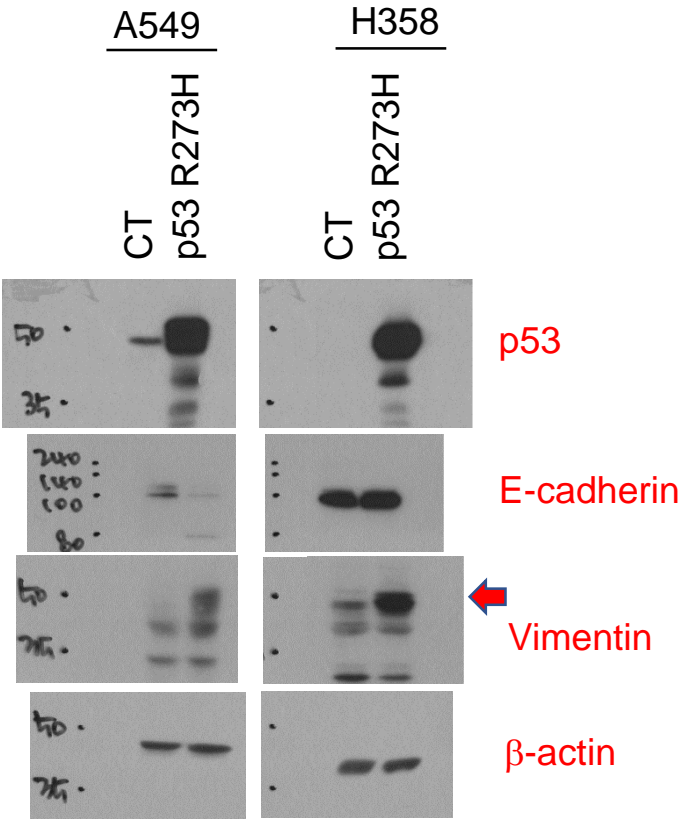

Supplement: Supplementary file 1 — Supplementary Information 1. [file 41598_2021_99267_MOESM1_ESM.pdf]
