# Supplementary material for: Contribution of p53 in sensitivity to EGFR tyrosine kinase inhibitors in non-small cell lung cancer
Source: Sci Rep. 2021 Oct 4;11:19667. doi: 10.1038/s41598-021-99267-z (PMC8490392; doi:10.1038/s41598-021-99267-z)
Supplement: Supplementary file 2 — Supplementary Information 2. [file 41598_2021_99267_MOESM2_ESM.docx]

**Supplemental information**

**Contribution of p53 in sensitivity to EGFR tyrosine kinase inhibitors in non-small cell lung cancer**

Authors: Sangyong Jung^1, *^, Dong Ha Kim^2, *^, Yun Jung Choi^2^, Seon Ye Kim^2^, Hyojeong Park^1^, Hyeonjeong Lee^1^, Chang-Min Choi^3^, Young Hoon Sung^4^, Jae Cheol Lee^5, †^, Jin Kyung Rho^4, †^

^1^Department of Biomedical Sciences, Asan Medical Center, AMIST, University of Ulsan College of Medicine, Seoul 05505, Republic of Korea

^2^Asan Institute for Life Sciences, Asan Medical Center, University of Ulsan of Medicine, Seoul 05505, Republic of Korea

^3^Department of Pulmonology and Critical Care Medicine, Asan Medical Center, University of Ulsan College of Medicine, Seoul 05505, Republic of Korea

^4^Department of Convergence Medicine, Asan Medical Center, University of Ulsan College of Medicine, Seoul 05505, Republic of Korea

^5^Department of Oncology, Asan Medical Center, University of Ulsan College of Medicine, Seoul 05505, Republic of Korea

^*^These authors contributed equally to this work

^†^Corresponding author:

Jae Cheol Lee

Department of Oncology, University of Ulsan College of Medicine, 88, Olympic-ro 43-gil, Songpa-gu, Seoul 05505, Republic of Korea. Tel.: +82 2 3010 3208; Fax: +82 2 3010 6961; E-mail: jclee@amc.seoul.kr

Jin Kyung Rho

Department of Convergence Medicine, University of Ulsan College of Medicine, 88, Olympic-ro 43-gil, Songpa-gu, Seoul 05505, Republic of Korea. Tel.: +82 2 3010 2974; Fax: +82 2 3010 6961; E-mail: jkrho@amc.seoul.kr


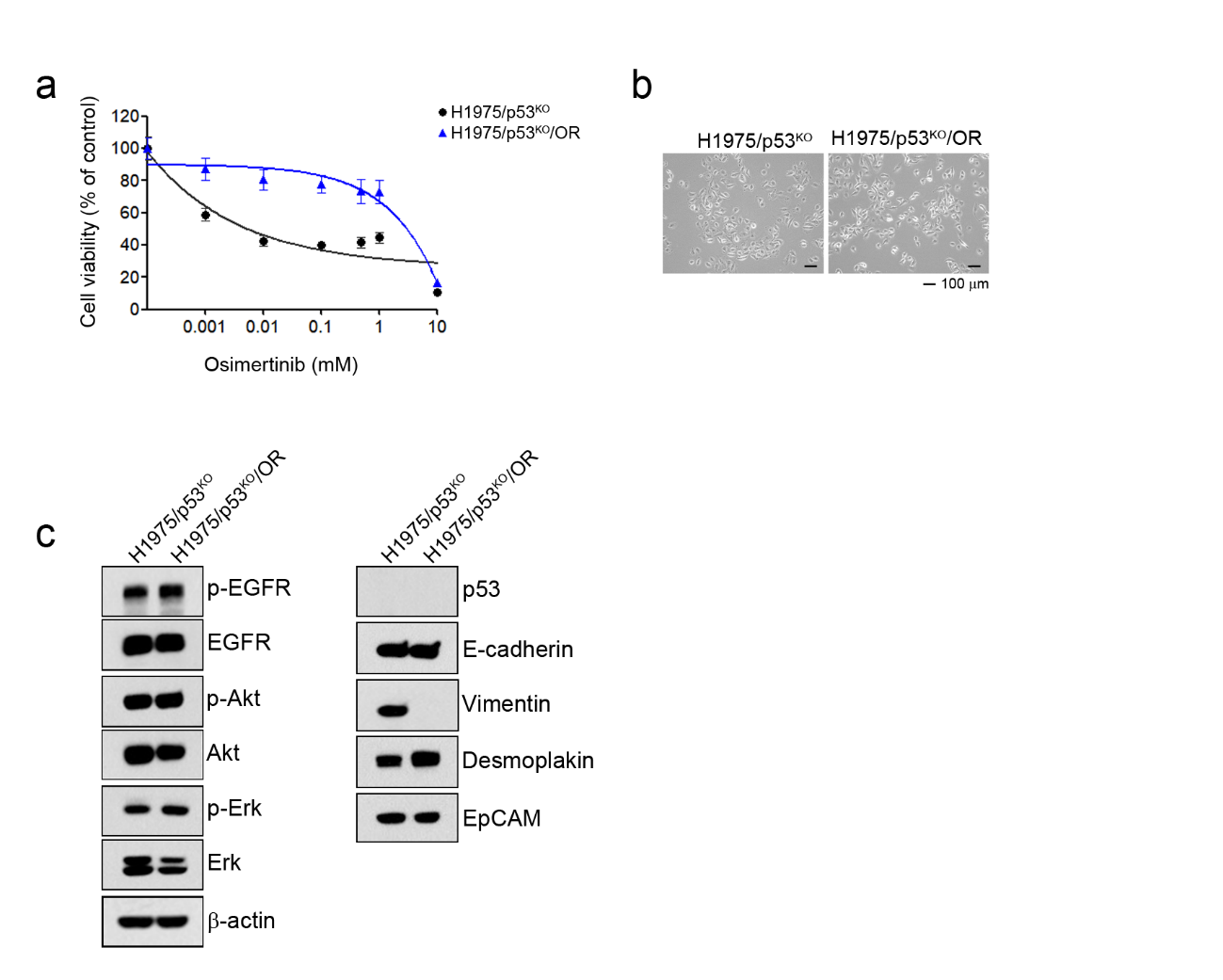


**Supplemental Figure S1. The characterization of H1975/p53^KO^/OR.**

Osimertinib-resistant cells (H1975/p53^KO^/OR) were established as described in the Materials and Methods section. These resistant cells were treated with osimertinib for 6 months. (a) Cells were treated with the indicated doses of osimertinib for 72 h, and cell viability was determined using MTT assays. (b) Cells were evaluated for morphologic changes using light microscopy. (c) EGFR-related signaling proteins and EMT-related proteins were analyzed by immunoblotting.


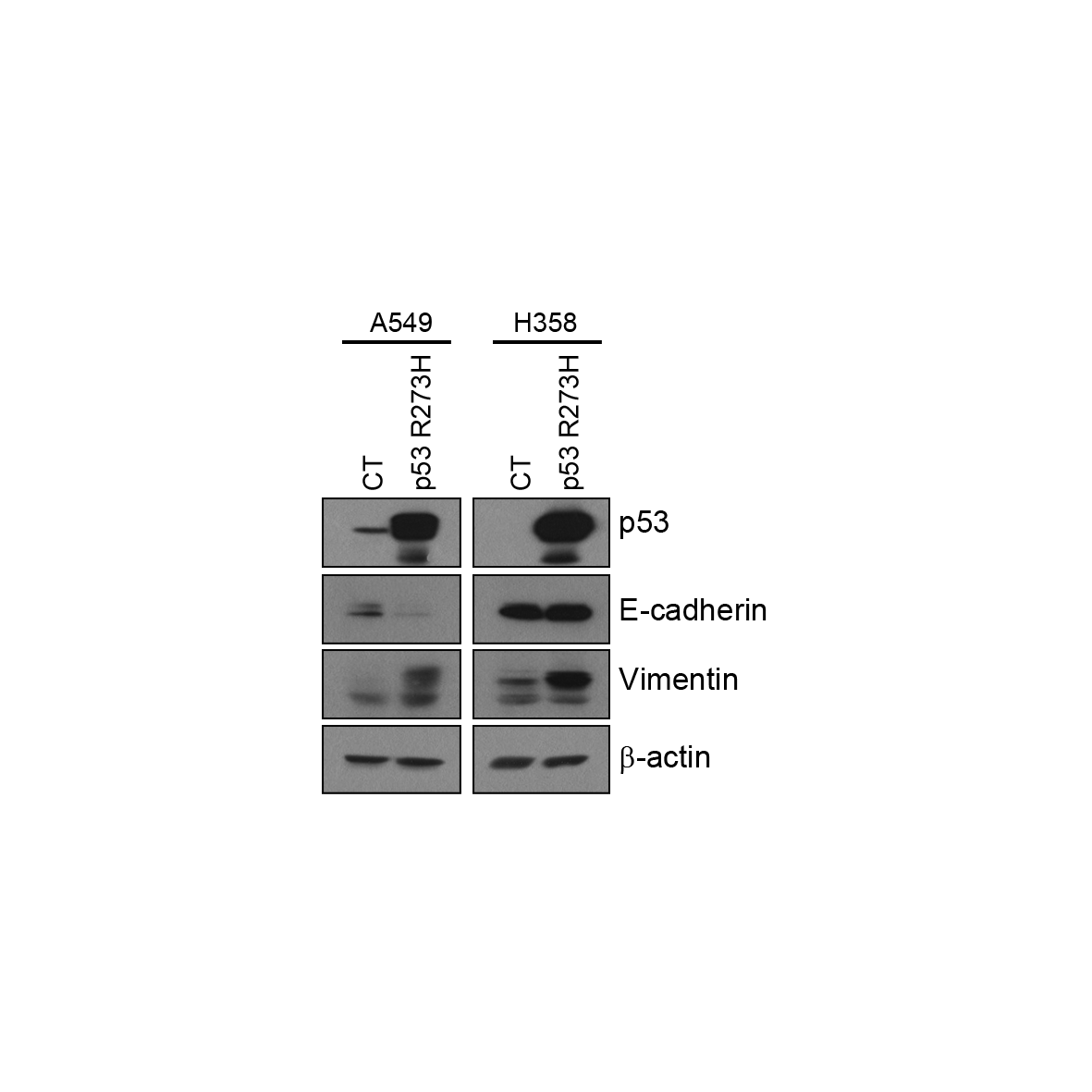


**Supplemental Figure S2. EMT induction by p53-R273H in NSCLC cells.**

Mutant p53-R273H was introduced into A549 (wild-type p53) and H358 (p53 null) cells; p53 and EMT-related protein levels were analyzed by immunoblotting.
